# Supplementary material for: The Ang-(1–7)/MasR axis ameliorates neuroinflammation in hypothermic traumatic brain injury in mice by modulating phenotypic transformation of microglia
Source: PLoS One. 2024 May 10;19(5):e0303150. doi: 10.1371/journal.pone.0303150 (PMC11086881; doi:10.1371/journal.pone.0303150)
Supplement: S1 Raw data — (DOCX) [file pone.0303150.s002.docx]

**S2_Raw_data**

**Table 1.** Quantitative RT-qPCR analysis of BV2 cells showing mRNA expression of IL-1β, IL-6, IL-4, and IL-10 after processing (value, Mean ± SD).

| Groups | N | Fig 1 | | | |
| --- | --- | --- | --- | --- | --- |
|  |  | A | B | C | D |
| TS | 3 | 106.92 ± 13.88^**^ | 3504.07 ± 248.68^**^ | 1.81 ± 0.71^*^ | 7.25 ± 0.22^**^ |
| TS+Ang-(1-7) | 3 | 66.83 ± 8.31^##^ | 1492.21 ± 162.53^##^ | 2.61 ± 0.27^#^ | 9.65 ± 2.93^#^ |
| TS+Ang-(1-7)+A779 | 3 | 105.99 ± 20.99^&&^ | 2045.97 ± 301.86^&&^ | 1.03 ± 0.30^&&^ | 7.18 ± 0.46^&^ |
| F value |  | 56.44 | 187.00 | 13.94 | 24.80 |
| P value |  | 0.000 | 0.000 | 0.000 | 0.000 |

Data are expressed as fold change compared to the control group.

Compared to the control group: ^*^P < 0.05, ^**^P < 0.01. Compared to the TS group: ^#^P < 0.05, ^##^P < 0.01. Compared to the TS+Ang-(1-7) group: ^&^P < 0.05, ^&&^P < 0.01.

**Table 2.** Western blotting assay and quantification showing protein expression of IL-6 and IL-10 in microglia after processing (value, Mean ± SD).

| Groups | N | Fig 1 | | |
| --- | --- | --- | --- | --- |
|  |  | F | G | H |
| TS | 3 | 1.75 ± 0.07^**^ | 1.17 ± 0.01^**^ | 1.32 ± 0.05^**^ |
| TS+Ang-(1-7) | 3 | 0.90 ± 0.23^##^ | 1.29 ± 0.06^**#^ | 1.79 ± 0.04^**^^##^ |
| TS+Ang-(1-7)+A779 | 3 | 1.33 ± 0.08^&&^ | 0.27 ± 0.08^&&^ | 1.32 ± 0.07^&&^ |
| F value |  | 28.27 | 242.11 | 138.30 |
| P value |  | 0.000 | 0.000 | 0.000 |

Data are expressed as fold change compared to the control group.

Compared to the control group: ^*^P < 0.05, ^**^P < 0.01. Compared to the TS group: ^#^P < 0.05, ^##^P < 0.01. Compared to the TS+Ang-(1-7) group: ^&^P < 0.05, ^&&^P < 0.01.

**Table 3.** Double staining of CD86 (red)/CD206 (green) in microglia of each group (value, Mean ± SD).

| Groups | N | Fig 2 | |
| --- | --- | --- | --- |
|  |  | B | C |
| TS | 6 | 89.06 ± 57.84^**^ | 34.00 ± 12.94^*^ |
| TS+Ang-(1-7) | 6 | 23.22 ± 21.08^##^ | 67.20 ± 48.88^#^ |
| TS+Ang-(1-7)+A779 | 6 | 79.54 ± 30.78^&^ | 21.88 ± 18.42^&&^ |
| F value |  | 9.30 | 6.36 |
| P value |  | 0.000 | 0.003 |

Data are expressed as fold change compared to the control group.

Compared to the control group: ^*^P < 0.05, ^**^P < 0.01. Compared to the TS group: ^#^P < 0.05, ^##^P < 0.01. Compared to the TS+Ang-(1-7) group: ^&^P < 0.05, ^&&^P < 0.01.

**Table 4.** Western blotting assay and quantitative expression of markers, cytokines, and MasR in microglia of each group (value, Mean ± SD).

| Groups | N | Fig 2 | | | | |
| --- | --- | --- | --- | --- | --- | --- |
|  |  | E | F | G | H | I |
| TS | 3 | 1.48 ± 0.08^**^ | 1.70 ± 0.62^*^ | 1.78 ± 0.07^**^ | 1.69 ± 0.09^**^ | 1.24 ± 0.08^**^ |
| TS+Ang-(1-7) | 3 | 1.25 ± 0.06^##^ | 2.68 ± 0.21^##^ | 1.50 ± 0.07^##^ | 1.99 ± 0.15^#^ | 1.12 ± 0.06^*#^ |
| TS+Ang-(1-7)+A779 | 3 | 1.59 ± 0.14^&&^ | 1.01 ± 0.05^&&^ | 1.12 ± 0.06^&&^ | 1.33 ± 0.18^&&^ | 0.92 ± 0.06^&&^ |
| F value |  | 27.71 | 17.56 | 120.33 | 36.44 | 24.80 |
| P value |  | 0.000 | 0.001 | 0.000 | 0.000 | 0.001 |

Data are expressed as fold change compared to the control group.

Compared to the control group: ^*^P < 0.05, ^**^P < 0.01. Compared to the TS group: ^#^P < 0.05, ^##^P < 0.01. Compared to the TS+Ang-(1-7) group: ^&^P < 0.05, ^&&^P < 0.01.

**Table 5.** The proportion of regional normal neurons and the proportion of Iba-1-positive cells in the region (value, Mean ± SD).

| Groups | N | Fig 3 | |
| --- | --- | --- | --- |
|  |  | B | C |
| Control | 3 | 6.82 ± 0.37 | 0.28 ± 0.01 |
| TS | 3 | 4.85 ± 0.64 | 1.46 ± 0.24 |
| TS+Ang-(1-7) | 3 | 6.20 ± 0.82^#^ | 0.99 ± 0.17^#^ |
| TS+Ang-(1-7)+A779 | 3 | 3.88 ± 0.08^**&&^ | 1.46 ± 0.27^**&^ |
| F value |  | 17.32 | 23.55 |
| P value |  | 0.001 | 0.000 |

Compared to the control group: ^*^P < 0.05, ^**^P < 0.01. Compared to the TS group: ^#^P < 0.05, ^##^P < 0.01. Compared to the TS+Ang-(1-7) group: ^&^P < 0.05, ^&&^P < 0.01.

**Table 6.**  Crossing numbers.(value, Mean ± SD).

| Groups | N | Fig 4B | | | | |
| --- | --- | --- | --- | --- | --- | --- |
|  |  | Before | Day 1 | Day 3 | Day 7 | Day 14 |
| Control | 3 | 135.47 ± 13.07 | 132.78 ± 19.88 | 122.86 ± 13.89 | 126.34 ± 7.38 | 125.92± 29.63 |
| TS | 3 | 134.57 ± 10.74 | 73.88 ± 30.88 | 83.33 ± 42.13 | 89.57 ± 31.06 | 99.27 ± 26.45 |
| TS+Ang-(1-7) | 3 | 137.14 ± 6.68 | 102.53 ± 30.75 | 117.31 ± 25.16 | 131.44 ± 21.55 | 136.81 ± 4.35 |
| TS+Ang-(1-7)+A779 | 3 | 138.37 ± 10.16 | 69.76 ± 23.18 | 92.97 ± 6.92 | 114.31 ± 18.64 | 109.29 ± 9.42 |
| T value |  | 52.41 | 9.42 | 12.94 | 16.08 | 17.62 |
| P value |  | 0.000 | 0.000 | 0.000 | 0.000 | 0.000 |

**Table 7.**  Rearing numbers.(value, Mean ± SD).

| Groups | N | Fig 4C | | | | |
| --- | --- | --- | --- | --- | --- | --- |
|  |  | Before | Day 1 | Day 3 | Day 7 | Day 14 |
| Control | 3 | 29.67 ± 4.04 | 24.67 ± 11.02 | 24.67 ± 3.06 | 29.00 ± 8.19 | 27.00± 5.00 |
| TS | 3 | 31.00 ± 13.00 | 10.33 ± 5.51 | 15.33 ± 11.55 | 21.33 ± 14.19 | 22.33 ± 13.05 |
| TS+Ang-(1-7) | 3 | 31.33 ± 13.01 | 26.00 ± 12.49 | 27.67 ± 12.50 | 30.00 ± 10.54 | 29.00 ± 4.58 |
| TS+Ang-(1-7)+A779 | 3 | 30.67 ± 14.74 | 18.00 ± 2.00 | 19.67 ± 2.08 | 24.67 ± 11.02 | 24.67 ± 4.16 |
| T value |  | 10.40 | 6.88 | 8.49 | 8.91 | 12.71 |
| P value |  | 0.000 | 0.000 | 0.000 | 0.000 | 0.000 |

**Table 8.** mNSS and Garcia score (value, Mean ± SD).

| Groups | N | Fig 4 | |
| --- | --- | --- | --- |
|  |  | D | E |
| Control | 3 | 1.00 ± 1.00 | 20.67 ± 0.58 |
| TS | 3 | 11.00 ± 1.00 | 11.67 ± 0.58 |
| TS+Ang-(1-7) | 3 | 9.00 ± 1.00^#^ | 14.00 ± 1.00^##^ |
| TS+Ang-(1-7)+A779 | 3 | 11.67 ± 1.15^**&^ | 12.00 ± 1.00^**&^ |
| F value |  | 66.77 | 78.79 |
| P value |  | 0.000 | 0.000 |

Compared to the control group: ^*^P < 0.05, ^**^P < 0.01. Compared to the TS group: ^#^P < 0.05, ^##^P < 0.01. Compared to the TS+Ang-(1-7) group: ^&^P < 0.05, ^&&^P < 0.01.

**
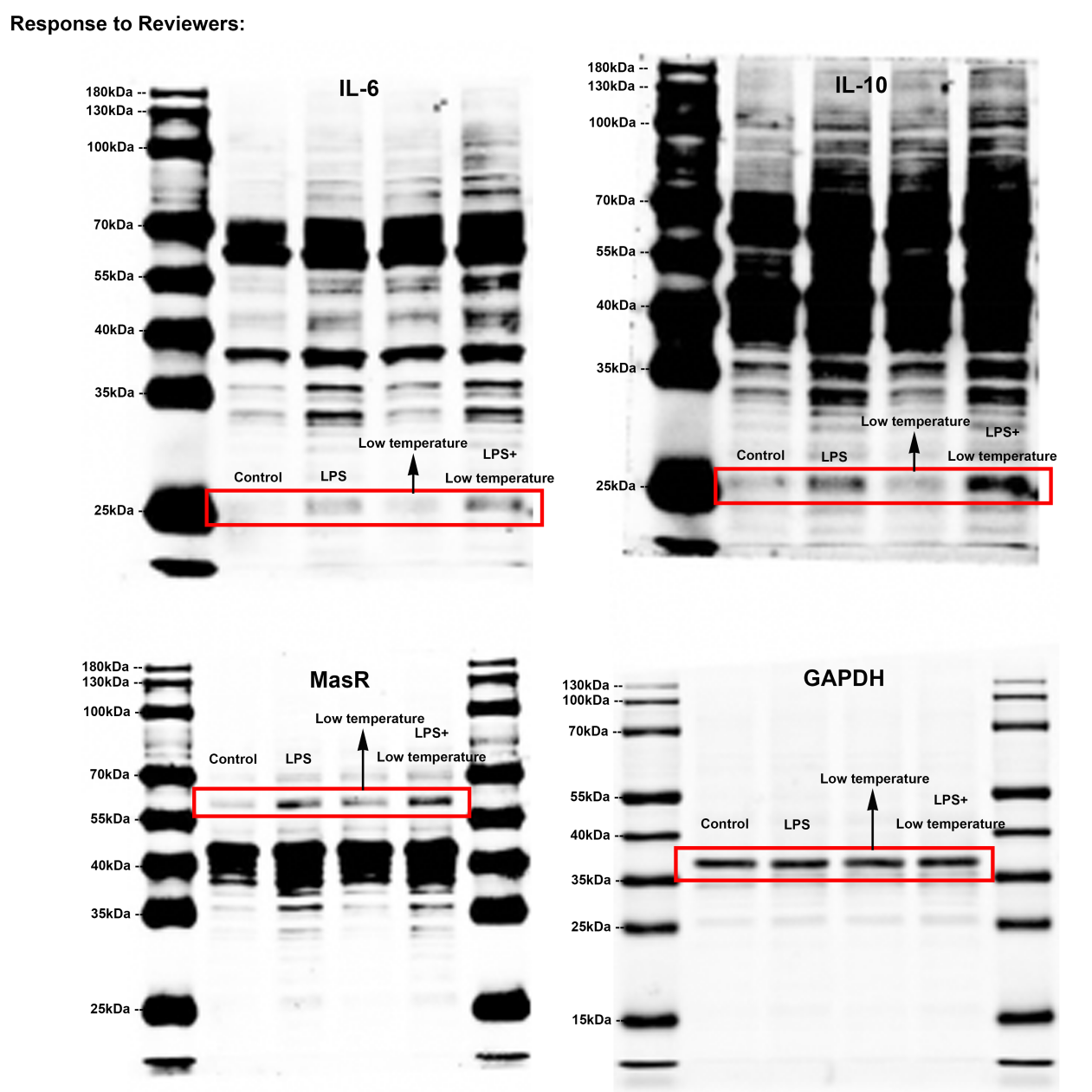
**
