# Supplementary material for: The Ang-(1–7)/MasR axis ameliorates neuroinflammation in hypothermic traumatic brain injury in mice by modulating phenotypic transformation of microglia
Source: PLoS One. 2024 May 10;19(5):e0303150. doi: 10.1371/journal.pone.0303150 (PMC11086881; doi:10.1371/journal.pone.0303150)
Supplement: Attachment — Submitted filename: Supplementary response to reviewers.docx [file pone.0303150.s004.docx]

**Dear Editor,**

Thank you very much for your replies and all reviewers’ kindly comments of our manuscript entitled **“The Ang-(1-7)/MasR axis ameliorates neuroinflammation in hypothermic traumatic brain injury in mice by modulating phenotypic transformation of microglia” (PONE-D-23-41383).** We apologise that some of the details in the manuscript have confused you. We have carefully considered your comments and supplemented with sources of relevant antibodies in Materials and Methods. We selected the bands by the size of the molecular weight of the samples in the specification, and the selected bands are all located within the example range in the specification. Following are the instructions for the antibodies used in the Western blot.

As for the band of MasR, we apologize for the confusion caused by the inappropriate labelling. In the letter to the reviewers, the MasR we labelled seems to deviate from his own molecular weight size, but a look at the whole figure reveals that the bands in either interval express the same tendency, i.e., that hypothermia affects the Ang-(1-7)/MasR axis to a certain extent. Previously we repeated the experiment and got the same conclusion. We are sorry that the image we chose may not be clear enough, and this more visual figure is attached below to provide you an explanation, which we hope will help to answer your confusion.

We would like to take this opportunity to thank you for all your time involved and this great opportunity for us to improve the manuscript. We hope you will find this revised version satisfactory.

Thank you and best regards.

Yours sincerely.

Shousen Wang, MD, PhD

Department of Neurosurgery,

900th Hospital,

Fuzhou 350025, Fujian Province, China.

Tel: + (86)13950482966.

E-mail address: wshsen1965@126.com
